# Psychological Benefits of Nonpharmacological Methods Aimed for Improving Balance in Parkinson's Disease: A Systematic Review

**DOI:** 10.1155/2015/620674

**Published:** 2015-07-07

**Authors:** Rastislav Šumec, Pavel Filip, Kateřina Sheardová, Martin Bareš

**Affiliations:** ^1^International Clinical Research Center (ICRC), St. Anne's University Hospital, Pekařská 53, 656 91 Brno, Czech Republic; ^2^First Department of Neurology, Faculty of Medicine, Masaryk University, St. Anne's University Hospital, Pekařská 53, 656 91 Brno, Czech Republic; ^3^Central European Institute of Technology (CEITEC), Behavioural and Social Neuroscience Research Group, Kamenice 753/5, 625 00 Brno, Czech Republic; ^4^Department of Neurology, Medical School, University of Minnesota, Minneapolis, MN 55455, USA

## Abstract

Parkinson's disease (PD) is a serious condition with a major negative impact on patient's physical and mental health. Postural instability is one of the cardinal difficulties reported by patients to deal with. Neuroanatomical, animal, and clinical studies on nonparkinsonian and parkinsonian subjects suggest an important correlation between the presence of balance dysfunction and multiple mood disorders, such as anxiety, depression, and apathy. Considering that balance dysfunction is a very common symptom in PD, we can presume that by its management we could positively influence patient's state of mind too. This review is an analysis of nonpharmacological methods shown to be effective and successful for improving balance in patients suffering from PD. Strategies such as general exercise, robotic assisted training, Tai Chi, Qi Gong, Yoga, dance (such as tango or ballet), box, virtual reality-based, or neurofeedback-based techniques and so forth can significantly improve the stability in these patients. Beside this physical outcome, many methods have also shown effect on quality of life, depression level, enjoyment, and motivation to continue in practicing the method independently. The purpose of this review is to provide information about practical and creative methods designed to improve balance in PD and highlight their positive impact on patient's psychology.

## 1. Introduction

Parkinson's disease (PD) is a progressive neurodegenerative disorder resulting in multiple motor and nonmotor symptoms [1]. The primary symptoms are caused by reduced activity of dopaminergic cells in substantia nigra [2]. Additionally, other motor, associative, orbitofrontal, and limbic circuits, which connect other brain's areas to basal ganglia, are affected as well [3, 4]. The disease is considered idiopathic, if occurring after the age of 50; however, some case can be attributed to mutation of several specific genes [5].

Cardinal motors symptoms can be merged under the acronym* TRAP*, which goes for tremor, rigidity, akinesia (bradykinesia), and postural instability [1]. Tremor is the most apparent and well known motor symptom. In most cases it is a rest tremor (maximal when limb is at rest), unilateral, and most prominent on the distal part of the limb and it disappears with an action or during sleep. Rigidity is caused by an excessive and continuous muscle contraction [1]. Just like tremor, it is often asymmetrical and is a usual cause of arthralgia, which may even be an initial sign of this disease [6]. Akinesia (or bradykinesia) represents slowness and difficulties around the whole movement process, regarding planning, initiation, and execution of movement. This symptom is especially problematic in terms of daily tasks such as getting dressed, writing, or sewing. Patients with PD usually have less difficulties with movement when some kind of external cue is provided [1, 7]. Postural instability and susceptibility to falls are other very serious and common symptoms occurring in PD, which even worsen over time [8, 9]. Balance dysfunction can be measured by multiple tests and scales (see Table 1).

Balance dysfunction has been identified as the main motor determinant of poor quality of life (QoL) [72–74]. In a particular falls, shuffling, and difficulty turning resulting from a postural instability are reported to be problematic [75]. Approximately 70% of patients report falls, often resulting in serious consequences such as fractures [76] or social stigma caused by embarrassment of falling in public [75]. Fear of falling can prevent patients from attending outdoor activities, which further reduces QoL. Performance in Timed Up and Go test (simple test requiring both static and dynamic balance) showed to be one of the important predictors of QoL [77]. Improvement of balance is considered to be one of the most relevant outcomes necessary for considering the treatment effective and successful [78]. Other important motor factors contributing to overall life quality are impairment of gait and adverse effects of patient's medication [79].

Nonmotor symptoms are represented by disorders of cognition, thoughts, mood, behaviour, and speech [1]. Cognitive impairments result especially in executive dysfunction, attention deficits, slow cognitive speed, recalling learned information, and visuospatial problems [80]. Most frequent mood difficulties are depression, anxiety, and apathy [1]. Depressive symptoms are present in 30–50% of PD patients. On one hand, they may be a psychological reaction to the disease; on the other hand, sometimes they occur even before the motor signs [81]. Depression provides the largest contribution to health related QoL, followed by postural instability, gastrointestinal symptoms, and urinary symptoms [82]. Anxiety has only recently attracted scientific attention, despite its high prevalence and a major impact on daily functioning and QoL. It is even reported as a significant factor resulting in deterioration of parkinsonian symptomatology [83, 84]. Approximately 45% of PD patients experience symptoms of anxiety. Anxiety related diseases, such as social phobia, generalized anxiety disorder, and anxiety disorder not otherwise specified (NOS) are most often diagnosed in this population [84–86]. Apathy is a frequent psychiatric disturbance that can manifest even earlier than the motor symptoms of PD [87]. It is diagnosed in 20–36% of new-onset PD patients [88, 89]. It has been shown to be related to more severe motor symptoms and higher risk of dementia development [90, 91]. Psychotic symptoms, such as hallucination and delusions, occur in 4% of patients and are often associated with dopaminergic excess caused by the treatment [92].

### 1.1. Balance and Mood Disorders

Postural instability is strongly associated with all cardinal mood disorders, such as anxiety, depression, and apathy [93, 94]. Anxiety, being the most researched mood disorder in relation to balance showed to be correlated with it in multiple neuroanatomical, experimental, and clinical studies.

**Figure 1:**
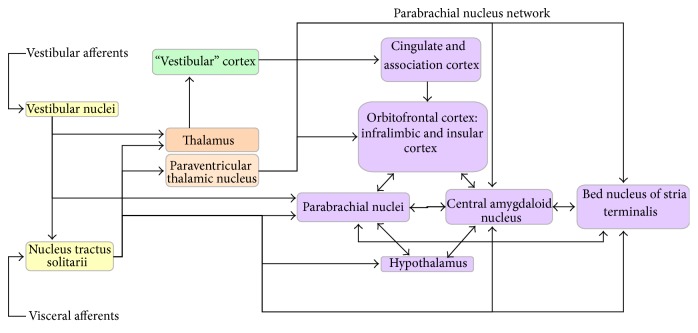
Neural balance-anxiety links.

Anxiety and balance share some common neural circuits [95, 96] (see Figure 1). Central circuits that process afferent visceral and vestibular information related to balance control include vestibular nuclei, nucleus tractus solitarii, thalamus, and vestibular cortex. These pathways connect to parabrachial nucleus network, which is involved in generating emotional and physiological manifestations of anxiety and fear [97].

Vestibular nuclei and nucleus tractus solitarii send ascending neurons to prosencephalon. Nucleus tractus solitarii sends axons directly to amygdala, thalamus, basal forebrain (bed nucleus of the stria terminalis), hypothalamic nuclei [98, 99], and the paraventricular thalamic nucleus. Paraventricular thalamic nucleus sends neurons to multiple regions, involving central amygdaloid nucleus, bed nucleus of the stria terminalis, and infralimbic cortex [100]. Neural tracts connecting the vestibular nuclei to the parabrachial nucleus are especially important. They represent a direct link between the vestibular networks and circuits involved in manifestation of anxiety and emotions [97, 101, 102]. Parabrachial nucleus therefore represents a junction for ascending vestibular and visceral information to basal forebrain, hypothalamic, amygdaloid, and these cortical regions construct an integral part of neural links that mediate anxiety and fear [97, 103].

An existing link between balance and anxiety has already been reported in studies using animal models. Mutant vestibular Headbanger (HdB) mice (with mutation resulting in imbalance) demonstrate progressive balance disability and elevated anxiety, when compared to wild type mice. After attending a balance training, HdB mice improved their balance and decreased the level of anxiety. Balance dysfunction therefore seems to have a causal effect on anxiety. Therefore improving it may be the right treatment for some anxiety disorders [104].

This causal hypothesis has also been tested on 20 healthy adults with the same results. After attending Balance and Reform program, their anxiety has significantly decreased and the level of self-esteem has significantly increased [105]. Study with children confirmed that children suffering from balance dysfunction had higher anxiety and lower self-esteem than healthy ones. Also in this case, the nonpharmacological balance training not only improved their balance, but also reduced anxiety and increased self-esteem when compared to the control group, which did not attend the exercise [106]. Detailed questionnaires and balance tests confirmed an excessive sensitivity of anxiety disordered children to balance-challenging situations [107].

Clinical studies with patients suffering from PD also suggest that there might be a clinical correlate to the connection of neural circuits responsible for balance and anxiety. A systematic review concerning factors associated with depression and anxiety among adults with PD mentioned that they seem to influence multiple motor or nonmotor symptoms [108]. It has been revealed that PD patients with balance impairment and gait difficulties were more likely to feel anxiety than patients whose dominant symptom was tremor [86]. Anxiety also seems to be associated with gait problems in PD [109] and is considered be a crucial mechanism underlying freezing of gait [110]. It has also been found in both PD patients and controls that the balance can be equally altered by anxiety, supporting the connection between neural links controlling emotion and stability [94].

Depression and apathy in terms of their connection to postural instability have been the major focus of limited number of studies. Although both symptoms are well characterized anatomically and are known to share deficits in cingulate and prefrontal cortex [111–113], their neuroanatomical basis for postural instability is less clear. However, it is known that depression, apathy, and balance all share basal ganglia circuitry and the dysfunction of dopamine system [111–114]. It has been shown that especially non-tremor-dominant PD patients have problems with these mood disorders [115]. It has also been observed that in early stages of PD, gait impairment is often associated with mild depressive symptoms [116]. In a statistical analysis of demographic, clinical, depression, and apathy data of patients with PD, apathy and depression have been associated with postural instability. It has been suggested that incidental postural instability should be of high concern in management of these mood disorders. Future research is necessary to better assess the pathophysiology and treatment of depression, apathy, and balance dysfunction [93].

### 1.2. Balance and Medication Treatment Limitations

Even though there is no cure for PD, medication, surgery, and palliative care can provide relief from some symptoms. However, pharmacological treatment hardly influences balance dysfunction. Treatment with levodopa even increases postural sway abnormalities [117].

It would therefore be very beneficial to find and apply more alternative, nonpharmacological solutions for treating balance dysfunction in patients with PD. There are multiple methods for improving balance of PD patients and the purpose of this review is to increase the awareness of this choice as a potential way to positively affect patient's mind and improve their QoL too.

## 2. Materials and Methods

In February 2015, we systematically searched PubMed, Web of Science, and EBSCO for articles pertaining nonpharmacological methods for improving balance in PD patients. We used the following keywords: Parkinson's disease, nonpharmacological, alternative, balance, instability, posture, and axial. The search of electronic databases identified 2160 articles. Titles and abstracts were reviewed to meet following inclusion criteria: (1) studies done on PD patients, (2) nonpharmacologically based, (3) aimed for improving balance, (4) published in last 20 years (1995–2015 (February)), and (5) full-text articles. After the reviewing process, 72 articles met the inclusion criteria. In the next step, full papers were analyzed with concern to study design and methodology. Following data were analyzed from the included studies: study characteristics (names of authors), training method, class of evidence, study design, duration of trial period, sample size, types of outcomes assessed, and the effect of the intervention. Any disagreement was resolved through discussion. After the reviewing process, 8 articles have been excluded because of their small sample size. 64 remaining studies have been further analyzed and commented on in this review (for compend, see Table 1).

### 2.1. General Exercise and Physiotherapy

The most researched practically applied strategy to improve the stability in PD is general exercise and physiotherapy. Exercise is structured and planned physical activity improves specific aspects of physical fitness [118]. Models of rehabilitation use multiple compensatory strategies as therapeutic approach. Evidence shows that exercise can also benefit in terms of neuroplasticity and brain's ability to reconstruct [119]. Randomised control trial (RCT) on exercising patients with PD showed positive changes in functional axial rotation (FAR) and functional reach (FR) in comparison with the control group [10]. The exercise showed the ability to improve equilibrium and modest gains in knee flexion and extension strength [11]. In RCT, it has been showed that it also results in consistent trend towards lower fall rates, lower rates of injurious falls needing medical attention, and higher score in QoL questionnaire [13]. Physiotherapy has shown to be effective in improving balance regardless of PD subtype (tremor dominant or akinetic-rigid) [18].

Exercise has shown to improve balance regardless of focusing on aerobic component. The thing that seems to matter the most is the focus on motor coordination, balance training, flexibility, and strength [14]. A RCT examining specifically designed exercise balance programme targeting multiple balance domains showed to be much more efficient in improving balance, stability, and gain in comparison to simple upper limb exercise [15]. The addition of method called Kinesio taping (KT) has not shown to have any further effect on balance [19].

An important question is whether the exercise is also effective when applied at home, without any special equipment, making it more practical and accessible for PD patients to attend. Home exercise intervention has also been proven to be effective in terms of improvement of postural control and balance [16]. A study using before-after study design testing supervised exercises on measures of static and dynamic balance have also proved to be effective, especially in terms of maximum excursion of limits of stability (LOS) [17].

When comparing a single balance training with training combining balance and resistance in a RCT study, both approaches show positive impact on balance; however, the effect is larger in the combined training [12]. Resistance exercise showed not only to improve balance, but bradykinesia and QoL too [21]. Balance training seems to be motivational for its attendants since most of them report that they would recommend it to other people [20].

### 2.2. Treadmill

Treadmill has also shown a potential to improve balance and gait in PD. The possible basis for this improvement is related to proprioceptive sensory cues provided by belt movement of this device [120]. When applied to people with PD, a pilot study using before-after design for treadmill training resulted in improvement in multiple balance scales as well as in QoL questionnaire [23]. When comparing the efficiency of assisted weight bearing treadmill training and the treadmill training alone, results show an improvement in dynamic posturography, number of falls, balance scales, and gait tests equally without any superiority of one method over the other [27]. When comparing walking on a treadmill and walking overground, both methods resulted in the increase of preferred speed walking; however, only the treadmill group improved in the stride length at the preferred and maximal walking speed, as well as other static posturography tests [25]. In RCT, partial weight-supported treadmill gait training (PWSTT) showed to be much more effective in terms of balance and gait improvement than conventional gait training, suggesting it to be a better interventional choice than gait physiotherapy [24]. Incremental speed-dependent treadmill training also showed trends towards improvement of postural instability, dynamic balance, and fear of falling [26]. Using additional body load resulted in improvement of multiple motor and nonmotor factors related to QoL [28].

### 2.3. Robot Assisted Gait Training

Electromechanical devices used to assist individual's gait and stepping have also been suggested as a potential intervention. When comparing effects of robot-assisted gait training (RAGT) and conventional physiotherapy on walking, RAGT showed to improve walking more efficiently [29].

RAGT has been examined in a RCT on postural stability too, also showing a significant improvement of balance when compared to conventional physiotherapy group [30]. Robotic treadmill training proved to be useful not just in improving motor symptoms, functional mobility, and walking capacity, but also a transient improvement in QoL [31]. Robot-assisted training has therefore proved to have a potential to enhance the effect of conventional physiotherapy not only in terms of gait, but also of balance.

### 2.4. Tai Chi, Qi Gong, and Yoga

Considerable amount of studies examined the potential of multiple exercises inspired by famous eastern techniques, such as Tai Chi (TC), Qi Gong, and Yoga. In particular TC has recently gained a lot of attention as an attractive intervention. The practice of this martial art involves slow and controlled movements and a need to maintain different kinds of postures. Therefore it has been suggested as an effective way to manage balance dysfunction. In a pilot study on PD patients TC intervention showed improvement in multiple balance and gait scales and tests [32]. In a RCT study, TC training also improved multiple motor control abilities, such as direction control, maximum excursion, functional reach, knee extension, and flexion peak torque, when compared to the control (stretching) group [33, 34]. TC training also leads to better dynamic postural control during obstacle negotiation, meaning that PD patients could more effectively negotiate an obstacle from the position of quiet stance at a normal speed, which results from a better balance control [35]. TC has also shown the potential to significantly improve balance, but also a significantly decrease in experience of falls, when compared to the control group. However, some measurements such as UPDRS III scores and TUG did not differ in comparison to the control group [36]. These optimistic results are not shared by all the studies. One RCT study showed that 16 weeks of TC training were ineffective in either gait performance, nor gait initiation, or the reduction of parkinsonian disability [37].

Qi Gong is another interesting balance training tested on individuals with PD. This Chinese martial art involves systematic training exercise, coordinated rhythmic movements, different breath patterns, and meditation. This mindful exercise is also reported to be involved in neuroplasticity, brain reorganization, and its repair. When tested on PD individuals, this method showed to have a positive influence on postural stability and Parkinson-related falls [38].

Another interesting and only recently researched method for postural stability is Yoga. This exercise was chosen as an intervention because of its known potential to increase flexibility, balance, and muscle strength [121–123]. Following two successful case studies [124, 125], one RCT study on PD patients reported that, alongside effects on physical aspects, yoga has also shown to improve depression, anxiety and QoL [39, 40].

### 2.5. Virtual Reality

Virtual reality (VR) and visual feedback training are becoming more frequent subject of research as an intervention for improving balance deficits for different types of population [126, 127]. One of these methods is The Nintendo Wii (Wii). Wii Fit balance board analyses data from the user's movements and uses them to perform a specific action in the game. The game then sends visual feedback to the user using a sensory-enriched environment. In an experimental noncontrolled study on patients with PD, Wii showed improvements in many balance tests. Authors suggested Wii to be an effective balance programme for PD patients, which is also practical because it can easily be used at home [41]. Wii seems to be effective not only on balance, but on the gait and postural sway too. However, in one prospective cohort study, it did not show to improve the balance confidence [42]. Wii intervention showed to have positive short-term motor and nonmotor effects along with an increase of QoL in PD patients. Authors encourage further studies to determine also long-term benefits of Wii [43]. Another study showed positive effects of virtual reality dance exercise on balance, depression, and activities of daily living. In this study, the experimental group, using Nintendo Wii, tried to mimic character on TV monitor, dancing to their favorite songs. If they mimicked properly, they felt vibrations of the remote control and were praised by the game [49]. When comparing the effect of Wii-based training versus balance exercise therapy, both groups of patients showed an improvement in activities of daily living, with no additional advantages in either of techniques [44].

Based on these findings other researchers hypothesized that Kinect Adventures games would be feasible and safe for people with PD too. When tested in a single-group blinded trial, the games showed an improvement in multiple scales of balance and gait, concluding it to be another effective strategy for enhancing patient's stability [45]. Similar study supported this claim by reporting an improvement in both balance and gait in 3 out of 5 observed subjects. The method has been therefore reported as a feasible, affordable, and safe option for the rehabilitation. However, their results will have to be confirmed by a larger-scale study [46].

One interesting trial compared effects of VR-based balance training using Xbox Kinect and conventional physiotherapy on functional balance and postural control. VR group showed a significant improvement in certain balance measures and positive trends in multiple subscales of Limits of Stability and gait. One RCT study has showed that Xbox Kinect seems to have some therapeutic potential; however so far there is no strong evidence to confirm its advantages above the conventional approach [47].

When comparing VR-augmented balance training, conventional balance training, and untrained group, VR training shows an additional improvement in certain equilibrium scores [128]. However, similar study reported no superiority of augmented visual feedback training over the conventional technique. However, it still proved to be another feasible and safe strategy to improve balance [48].

### 2.6. Neurofeedback Training

Another inspiring technique is Neurofeedback Training (NFT). In this method a participant receives feedback of signals which represent his/her subconscious neural activities. By observing his/her brainwaves on a computer screen, it is possible to learn to control and change them. This training leads to a point when one is able to control their brainwaves subconsciously not only through the exercise, but in their everyday life too [129]. It is already being used for treating many brain disorders [130]. In PD, balance and gait are tasks requiring attention. Therefore by improving patient's attention it is possible to maintain the balance for longer period of time [131, 132]. It is known that there is a relationship between particular levels of performance and concrete patterns of cortical activity. By using NFT for recreating these patterns in patients with motor deficiencies, it might be possible to enhance their performance. When applied on PD patients in a RCT study, NFT showed an improvement in both static and dynamic balance [50].

Researchers have tried to prove the same point using audiobiofeedback. In this method patients received an auditory feedback modulated by their movement. It showed an improvement in balance. The training also appeared to have a positive impact on psychosocial aspects of PD and the depression level of patients. Patients reported a high satisfaction attending this training [51].

Other researchers claimed that audiobiofeedback has disadvantages regarding the fact that audio information is quite nonintuitive and therefore it is not possible for patients to react on it fast enough. They suggested more intuitive, vibrotactile neurofeedback stimulus, which has been reported to have much faster reaction time [52]. Vibrotactile NFT really showed an ability to improve balance and also a potential to reduce the number of falls [133].

### 2.7. Dance

Dance is a multidimensional activity offering auditory, visual, and sensory stimulation, musical experience, social interaction, memory, motor learning, and emotional perception, expression, and interaction [134]. Several uncontrolled studies report that people with PD are motivated to attend dance lessons regularly, to have a great compliance and even a tendency to continue their attendance after the study is finished [55, 62].

Tango, as one of the most researched dances in terms of balance in PD, is reported to be very helpful for improving QoL. It puts the training of balance and gait in environment necessary for social interaction and close work with a dancing partner [62]. A noncontrolled trial of short and intensive tango lessons showed to significantly improve balance, however, not the gait [61]. When comparing effects of tango and conventional exercise on healthy elders and elders with PD in a RCT study, only the PD tango group improved all measures of balance, falls, and gait. Moreover, the PD tango group was more confident about balance than the PD exercise group. Both groups have reported a lot of enjoyment of their tango experience and overall treatment satisfaction [63, 64].

However, some researchers ponder on possible disadvantages of tango, regarding its requirement to memorize exact movements and the fact that some patients might just not be comfortable performing a certain style and aesthetics which tango requires. They suggested more of an improvising dance, which may offer more accessible technique, while still keeping the functional and social benefits of tango. So-called Contact Improvisation (CI) dance directs the dancer's attention to nonverbal communication and sensation rather than to visible appearance or specific movement sequences. CI challenges to adapt to unpredictable movement coming from tactile interaction with the dancing partner. In one noncontrolled study, CI shows significant improvements in multiple balance scales and tests. Additionally, PD dancers have expressed a lot of enjoyment and a motivation to attend future CI classes [53].

An intensive trial of modern dance for 11 adults with early-to-middle stage PD also showed an improvement in Fullerton Advanced Balance Scale, however, not in Timed Up and Go test [54]. When comparing effects of partnered and nonpartnered dance on balance and mobility, both groups showed improvement in balance and walking. However, only partnered participants expressed more enjoyment from classes and an interest to continue with it in future [135]. Dance shows to have short-term benefits on motor functions of PD patients; however, in one uncontrolled study, no improvement is reported regarding Timed Up and Go test and semitandem test. Even though it shows to improve QoL not only in patients, but their caregivers [56], dance therapy positively affects patient's mood, lowers their apathy and depression levels, and improves their neuropsychological performance [57, 58]. Apart of physical benefits, PD patients attending dance classes report emotional and social benefit too [59].

An interesting dance for managing a balance dysfunction is ballet. In ballet one needs to concentrate on body alignment, posture, and the coordination of the whole body. It challenges dancer's strength, stability, and encourages to train different movement qualities and dynamics. In case of PD patients, it showed to improve stability and balance, however, not the posture. Also in this case, participants were very motivated and reported the ballet-lessons to be an important aspect of their lives [60].

### 2.8. Deep Brain Stimulation

Deep brain stimulation (DBS) is another nonpharmacological option that has been tested on balance dysfunction in PD patients. This invasive method is widely used for those patients with motor symptoms which cannot be further managed by medication therapy. Candidates for this procedure are very carefully chosen by multidisciplinary team (neurologist, neurosurgeon, neuroradiologist, and neuropsychologist). Because of higher risk of cognitive decline and affective disorders after implantation of DBS in patients who have already had cognitive or affective problems before the operation, candidates for DBS should be carefully psychologically tested for signs of dementia or major depression [136].

However, efficiency of DBS on balance is not entirely clear. While some studies report improvements in posturography measures and balance scales [65, 137], other report that DBS may even worsen patient's gait, stability, and risk of fall [138]. Inaccurate positioning of the stimulating electrode can result in stimulation-induced freezing [139]. If the current spreads to the substantia nigra or other adjacent regions, it can cause akinesia and negatively affect patient's walking abilities. A meta-analysis showed that, during the first year after the surgery, the effect of subthalamic nucleus DBS on postural stability roughly equals the preoperative effect of medication [140]. Another problem is the worsening of axial motor functions despite the DBS treatment in the long term [141–144]. It has been reported that 35% patients develop worsening of postural stability after 5 to 8 years after the procedure [142]. Stimulation of pedunculopontine nucleus (PPN) has been proposed as an alternative for PD patients with axial signs. It has been shown to be more effective on axial signs than stimulation of subthalamic nucleus. However, these findings still need to be replicated in a larger number of patients and there have not been yet done a study with a high class of evidence that analyzed this issue [145].

### 2.9. Other Techniques

Another interesting method is the aquatic therapy. When compared to the conventional balance training in a RCT, it showed to have a bigger effect on postural stability in PD patients [66]. When comparing a whole body vibration (WBV) and conventional physiotherapy on disturbances of balance and gait there was no evidence for the superiority of WBV [67]. In one quasiexperimental study, cycle ergometry also showed to have a significant positive effect not balance impairment and PD-related disability [68]. Comparing the effect of transcranial direct current stimulation (tDCS), physical training and the combination of the two on balance and gait in PD also brought interesting results. It was shown that even if there is no benefit of using tDCS alone, when combined with physical training it increases gait speed and improves balance even more significantly than when using physical training alone [69]. As one RCT study has shown, another successful way to improve balance is repetitive step training, a volitional and compensatory step program focused on stability and gait. It showed improvement in multiple tests examining the patient's balance, stability, and gait [70]. Movement strategy training (MST), as another proposed technique, teaches people with PD to use their frontal cortex to move faster, more easily, and safely by using their cognition. By using focused attention, external cues, visualization, and mental rehearsal, participants are trained in their general mobility. When compared with the usual exercise in a RCT, participants of MST group showed improvements on several outcome measures, including balance and gait tests. However, participants did not manage to maintain all these long-term improvements [71].

## 3. Discussion

Basically all methods analyzed in this review showed positive results in terms of balance. Out of 72 studies, 70 have shown positive results concerning balance and 24 reported positive influence on patient's well-being. Studies varied in terms of study design, training time, sample size, and the outcome measures. Generally, studies examining physiotherapy have had the highest class of evidence, 13 of them being RCT with large sample size. Three of physiotherapy-based studies reported a better QoL [13, 21, 22] and 1 of them declared participants motivation to continue [20]. Four out of 6 treadmill-based studies have been RCT with sample size varying from 22–40 participants, all of them being successful in improving postural stability. Psychological benefits have been reported in 2 of them [23, 28], both of them lacking control group. Robotic gait training was assessed by 3 studies, 2 of them being RCT with sample sizes between 34 and 41 participants. Improved QoL was observed in the third one without control group, studied on 70 PD patients [31]. Tai Chi was examined by 5 RCT applied on large sample sizes varying from 45 to 195 subjects and one noncontrolled applied on 12 patients only. However, the question on psychological effect of this method in PD is not answered yet. Yoga, on the other hand, showed a lot of positive effects on participant's mind, such as enjoyment, relaxation, lowering the anxiety, and depression level [39, 40]. Since there was only one RCT with sample size of 13 participants only, these results should be further confirmed on larger studies [39]. As for virtual reality-based techniques, studies varied in study design (5 RCT, 5 noncontrolled trials), examining samples of medium size (between 18 and 42 subjects) with consistent training time (between 4 and 7 weeks) and 2 studies reporting positive psychological impact on patient's depression and QoL [43, 49]. Neurofeedback proved to be effective and useful in 3 out of 3 studies, one of them being RCT with 16 patients [50], other two not controlled studies with sample size no bigger than 10 patients [51, 52]. Dance, especially tango, seems to have perhaps the most promising results in terms of both balance and psychological well-being. Out of 13 reviewed studies, 9 have reported participants satisfaction, emotional and social benefits and improvement in apathy and depression scales. 4 of 13 studies were RCT with large size groups (up to 75 participants), 8 were noncontrolled with medium size groups (between 8 and 39 participants) and one was a case study. Three studies examined the effect of DBS on balance, however, RCT assessing this issue has not been done yet, nor have they measured any psychological aspects related to better stability [137, 145, 146]. Other RCT testing techniques such as aquatic therapy [66], Whole Body Vibration [67], tDCS [69], and movement strategies [71], with group size varying between 11 and 28 participants, all showed promising results.

Some studies have been excluded from our analysis because of low level of evidence. Interesting ideas such as footwear modifications [147], box [148], and multidimensional exercise [149] are very compelling methods showing positive results in terms of both balance and patient's well-being. However, because of their small sample size, larger studies are necessary to prove their effectivity.

Even though there are many effective and creative methods that improve balance of patients with PD, they are not used that often due to some of their limitations. Because of an apathetic nature of PD, patients have low expectations that often limit their motivation to attend the method and keep their practice. Another important fact is that, in comparison to the pharmacological treatment, nonpharmacological methods are more time-consuming, making it difficult to include in everyday life. Some patients cannot comply simply because their fear of falling is too strong [150]. Another problem might be financial, such as high health care, medication, and travel cost, which sometimes make these therapies inaccessible and difficult for patients to maintain their participation [16].

## 4. Conclusions

The current review synthesized compelling evidence of efficiency of multiple methods proven to increase balance and stability in PD and highlights their potential for psychological benefit. Balance problems and psychological problems such as anxiety, depression, and apathy are very common in PD patients and all result in impaired QoL. Neuroanatomical [95], experimental, and clinical studies on animal models [104], children [106, 107], healthy young adults [105], and PD patients [86, 94, 108–110] support the hypothesis that balance and mental well-being are interconnected and should not be overlooked. Taking into consideration that pharmacological therapy has its limitations and comes with many side effects, employing scientifically proven strategies influencing balance and improving quality of life appears to be a reasonable choice to make.

## Figures and Tables

**Table 1:** Compend of studies examining different nonpharmacological strategies aiming to improve the balance in PD and their psychological benefits.

Author (citation)	Training method	Class of evidence	Training time	Sample size	Outcome measure	Balance improvement	Psychological benefits
*General exercise and physiotherapy *							
Schenkman et al. [10]	Exercise	1c	10 weeks	23 + 23	FAR, FR	Yes	
Toole et al. [11]	Balance and strength training program	1c	10 weeks		Equilibrium, knee flexion/extension strength	Yes	
Hirsch et al. [12]	Balance training and high-intensity resistance training	1c	10 weeks	15	SOT	Yes	
Ashburn et al. [13]	Exercise	1c 1c	8 weeks 8 weeks	70 + 72 70 + 72	FR, [lower fall rates] BBS, SPDDS	Yes No	QoL
Gobbi et al. [14]	Intensive exercise program Adaptive program	1c 1c	6 months 6 months	21 13	BBS, TUG BBS, TUG	Yes Yes	
Wong-Yu Is and Mak [15]	Balance exercise	1c	8 months	26 + 26	MiniBESTest, ABC, MDS-PIGD	Yes	
Nocera et al. [16]	Home-based exercise on postural control	1c	10 weeks	10 + 10	CDP, SOT	Yes	
Kara et al. [17]	Supervised exercise	2b	12 weeks	17	Balance Master protocol	Yes	
Frazzitta et al. [18]	Intensive rehabilitation treatment	2b	4 weeks	130	UPDRS II and III, SPDDS, BBS, 6 MWT	Yes	
Frazzitta et al. [18]	Balance training	2b	12 weeks	5	MiniBESTest	Yes	
Capecci et al. [19]	Postural rehabilitation Kinesio taping	1c 1c	4 weeks 4 weeks	(7 + 6) + 7 (7 + 6) + 7	BBS, TUG, trunk bending BBS, TUG, trunk bending	Yes Yes	
Conradsson et al. [20]	Progressive group balance training	2b	12 weeks	5	MiniBESTest	Yes	Motivated to continue
Dibble et al. [21]	High intensity eccentric resistance training	1c	12 weeks	10 + 10	UPDRS III, 10 MWT, TUG	Yes	QoL
Corcos et al. [22]	Progressive resistance exercise	1c	2 years	20 + 18	UPDRS III	Yes	QoL

*Treadmill *							
Herman et al. [23]	Treadmill	2b	6 weeks	9	PDQ-39, UPDRS III, WV, Swing time variability, SPPB	Yes Yes	QoL
Ganesan et al. [24]	Weight-supported treadmill	1c	4 weeks	20 + 20	UPDRS III, MLI, BBS, LOS, POMA	Yes	
Bello et al. [25]	Treadmill	1c	5 weeks	11 + 11	Preferred speed walking, SL at the preferred Maximal WV, TUG, Static posturography	Yes Yes	
Cakit et al. [26]	Speed-dependent treadmill	1c	8 weeks	21 + 10	Walking distance, Tolerated maximum speed, BBS, DGI, FES	Yes Yes	
Toole et al. [27]	Treadmill	1c	6 weeks	23	Dynamic posturography, falls, BBS, UPDRS III	Yes	
Filippin et al. [28]	Treadmill with additional body load	2b	18 weeks	9	UPDRS III	Yes	QoL

*Robotic gait training *							
Picelli et al. [29]	Robotic gait training	1c	3 weeks	21 + 20	10 MWST, 6 MWT	Yes	
Picelli et al. [30]		1c	4 weeks	17 + 17	BBS, NUTT	Yes	
Paker et al. [31]	Robotic treadmill training	2b	5 weeks	70	TUG test, 10 MWT, UPDRS	Yes	QoL

*Tai Chi*, *Qi Gong*, *and Yoga *							
Hackney and Earhart [32]	Tai Chi	1c	10–13 weeks	17 + 16	BBS, UPDRS, TUG, tandem stance test, 6 MWT, backward walking Forward walking, UST	Yes Yes No	
Tsang [33]		1c	24 weeks	65 + 65 + 65	Maximum excursion, Direction control, SL, FR, knee flexion/extension, TUG, UPDRS III, falls	Yes Yes	
Li and Fitzgerald [34]		1c	24 weeks	65 + 65 + 65	LOS (maximum excursion, directional control), SL, WV, FR, TUG, UPDRS III, RoF	Yes Yes	
Kim et al. [35]		2b	12 weeks	12	Dynamic postural stability	Yes	
Gao et al. [36]		1c	12 weeks	37 + 39	BBS, falls UPDRS III, TUG	Yes No	
Amano et al. [37]		1c	16 weeks	23 + 22	UPDRS III	No	
Loftus [38]	Qi Gong	2b	3 months	41	BBS, falls	Yes	
Sharma et al. [39]	Yoga	1c	12 weeks	8 + 5			QoL
Boulgarides et al. [40]		2b	8 weeks	10	SLB	Yes	HADS

*Virtual reality *							
Esculier et al. [41]	Nintendo Wii	2b	6 weeks	10 + 8	TUG, STST, UST, 10 MWT, CBM, POMA, force platform	Yes Yes	
Mhatre et al. [42]		2b	8 weeks	10	BBS, DGI, postural sway, Romberg with eyes closed Activities-specific Balance Confidence	Yes No	
Herz et al. [43]		2b	4 weeks	20	UPDRS III, TUG	Yes	QoL
Pompeu et al. [44]		1c	7 weeks	16 + 16	BBS, UST	Yes	
Pompeu et al. [45]	Kinect adventures	2b	5 weeks	7	6 MWT, BEST, DGI, PDQ-39	Yes	
Summa et al. [46]	Kinect	2b	5 weeks	5	TUG, 10 MWT, reduction of movement duration and curvature, movement acceleration	Yes Yes	
Shih et al. [47]	Xbox Kinect	1c	4 weeks	10	AT, [LOS, AT, TUG]	Yes	
Shih et al. [47]	Virtual reality-augmented balance training	1c	6 weeks	14 + 14 + 14	SOT Attentional demand for postural control	Yes No	
van den Heuvel et al. [48]	Augmented visual feedback	1c	5 weeks	17 + 16	[BBS, the single leg stance test, 10 MWT]	No	
Lee et al. [49]	Virtual reality dance exercise	1c	6 weeks	10 + 10	BBS	Yes	BDI

*Neurofeedback *							
Azarpaikan et al. [50]	Neurofeedback	1c	2, 5 weeks	8 + 8	Biodex, BBS	Yes	
Mirelman et al. [51]	Audiobiofeedback	2b	6 weeks	7	BBS, [TUG]	Yes	GDS, PDQ-39
Asseman et al. [52]	Vibrotactile neurofeedback	2b	2 weeks	10	Body sway, RoF, SOT, DHI, ABC	Yes	

*Dance *							
Marchant et al. [53]	Contact improvisation dance	2b	2 weeks	11	UPDRS III, BBS, increased swing and decreased	Yes	Reported enjoyment, motivated to continue
					Stance % during walking, backward step length	Yes	
Batson [54]	Modern dance	2b	3 weeks 3 weeks	11 11	FAB TUG	Yes No	
Hackney and Earhart [55]		2b	10 weeks	39	BBS, WV, cadence	Yes	Reported enjoyment, motivated to continue
Heiberger et al. [56]	Dance	2b	8 months 8 months	11 11	UPDRS III TUG, SeTa	Yes No	QoL
Rosario et al. [57]		2b	9 weeks	8	TUG	Yes	
Hashimoto et al. [58]		1c	12 weeks	46	TUG, BBS, FAB, UPDRS	Yes	AS, SDS
Westheimer et al. [59]		2b	8 weeks	12	UPDRS III	Yes	Reported emotional and social benefits
			8 weeks	12	BBS	No	
Houston and McGill [60]	Ballet	2b	12 weeks		FAB	Yes	
Hackney and Earhart [61]	Tango	2b	2 weeks	14	BBS, UPDRS III, time in stance during forward walking	Yes	
			2 weeks	14	TUG, 6 MWT	No	
Hackney and Earhart [62]		1c	13 weeks	19 + 19 + 19 + 18	QoL	Yes	QoL
Hackney et al. [63]		1c	12 weeks	19 + 19	ABC, Modified FES, FR, UST, WV	Yes	Reported enjoyment
Romenets et al. [64]		1c	12 weeks	18 + 15	Mini-BESTest, TUG time, [FSS]	Yes	Reported enjoyment, felt “overall” treatment satisfaction

*Deep brain stimulation *							
Nilsson et al. [65]	STN stimulation	2b		10	BBS, FES, posturography	Yes	

*Other methods *							
Vivas et al. [66]	Aquatic therapy	1c	2 weeks	6 + 5	FRT, BBS, UPDRS	Yes	
Ebersbach et al. [67]	Whole Body Vibration	1c	6 weeks	13 + 14	Tinetti Balance Scale, stand-walk-sit test,	Yes	
Lauhoff et al. [68]	Cycle ergometry	2b	6 weeks	23	BBS, TUG, ADL, UPDRS III.	Yes	
Kaski et al. [69]	Exercise + tDCS	1c	1 weeks	8 + 8	PT, WV	Yes	
Shen and Mak [70]	Repetitive step training	1c	4 weeks	6 + 6	Reaction time, movement velocity, LOS, UPDRS-PG	Yes	
Morris et al. [71]	Movement strategies	1c	2 weeks	14 + 14	UPDRS, 10 MWT, balance	Yes	

ABC: Activities-Specific Balance Confidence Scale; API: Anteroposterior Index; AS: apathy scale; AT: Adaptation Test; BBS: Berg Balance Scale; BDI: Beck Depression Inventory; BEST: Balance Evaluation Systems Test; CBM: Community Balance and Mobility Assessment; CDP: computerised dynamic posturography; DGI: Dynamic Gait Index; DHI: Dizziness Handicap Inventory; FAB: Fullerton Advanced Balance Scale; FAR: functional axial rotation; FES: Falls Efficacy Scale; FR: functional reach; HADS: Hospital Anxiety and Depression Scale; MDS-PIGD: Movement Disorder Society-Unified Parkinson's Disease Rating Scale-postural instability, gait difficulty score; LOS: limits of stability; MLI: Mediolateral Index; MiniBESTest: Minibalance Evaluation Systems Test; NUTT: Nutt's rating; OBI: Overall Balance Index; PDQ-39: Parkinson's Disease Questionnaire; POMA: Performance-Oriented Mobility Assessment; PT: Pull Test; QoL: quality of life; RoF: Rates of falling; SDS: Self-Rating Depression Scale; SeTa: semitandem test; SL: stride length; SOT: Sensory Organization Test; SPPB: Short Physical Performance Battery; SPDDS: Self-Assessment Parkinson's Disease Disability Scale; STST: Sit-to-Stand test; TUG: Timed Up and Go Test; UPDRS III.: Unified Parkinson's disease rating scale-Part III. Motor examination; UPDRS-PG: Unified Parkinson's disease rating scale-Posture and Gait; UST: Unipedal Stance Test; WV: Walking Velocity; 6 MWT: 6-Minute Walking Test; 10 MWST: 10-Minute Walking Speed Test; w: weeks; m: months; [  ]: trends.

## Compend of Anxiety Scales

BAI: Beck Anxiety Inventory
HADS: Hospital Anxiety and Depression Scale
SAS: Self-Rating Anxiety Scale
ASI: Anxiety Status Inventory
STAI: State Trait Anxiety Inventory
HARS: Hamilton Anxiety Rating Scale
MINI: Mini-International Neuropsychiatric Inventory (section for anxiety)
NPI: Neuropsychiatric Inventory (section E).
